# Biologicals acting against interleukin 1 may reduce corticosteroid dependence in systemic onset juvenile idiopathic arthritis

**DOI:** 10.1186/1546-0096-9-S1-P70

**Published:** 2011-09-14

**Authors:** W Emminger, A Ulbrich, E Pernetzky-Hintringer

**Affiliations:** 1 University ChildrenÂ´s Hosital of Vienna, Austria, St. Anna ChildrenÂ´s Hospital, Vienna, Austria

## 

Children suffering from systemic onset juvenile idiopathic arthritis (soJIA) are often receiving high doses of corticosteroids (CS) for a long time duration and therefore are suffering from many CS associated side effects. The therapy of soJIA includes non steroidal antiinflammatory drugs, CS and methotrexate +/- azathioprine. Other drugs like cyclosporin A, thalidomide and biologicals like tumor necrosis factor alpha-inhibitors or -antibodies have not been very successfully applied in many children. During the last years the interleukin-1 receptor antagonist anakinra and antibodies against interleukin 1ß have been developed and clinical experiences are showing promising antiinflammatory activity.

We present six patients with soJIA. The age at diagnosis was 2, 2, 3, 5, 10 and 15 years. During kineret therapy CS could be finished in five of them and is given in a very low dosage of 5 mg prednisolone daily in the sixth patient. The dose of kineret had to be individualized. In 5 patients the dose of kineret varied between 1,3 mg/kg and 4 mg/kg per day. Sometimes it had to be applied in two daily doses. In five patients kineret has been applied for 5, 8, 24, 29 and 45 months. In the sixth patient kineret has been applied for 15 months, and then the ILß antibody canakinumab has been applied for another 11 months in monthly intervals.

In May 2011 5/6 patients do not need any CS for 4,9,29,35 and 45 months, respectively. The sixth patient suffering from a very severe chronic inflammatory disease is receiving low doses of 5 mg prednisolone per day. Three of six patients are receiving additional low doses of weekly methotrexate, three patients do not need any immunosuppressive therapy at all.

Therapeutic drugs acting against interleukin 1 are promising therapeutic alternatives for patients suffering from soJIA. The subcutaneous applicability is another advantage. The duration of CS dependence may be reduced in many patients suffering from soJIA.

